# Causes of the Formation of Cataract

**Published:** 1868-01

**Authors:** Albert Mooren


ART. IV.—*Causes of the Formation of Cataract.* By Albert Mooren, M. D. *Translated from the German by* C. F. A. Nichell, M. D.

It has been at all times of the greatest interest to both physicians and laymen to investigate the causes of cataract formations. The old were pleased to search for the same in, and attribute them to the disturbance, the result of any accidental dyscrasia. A later age discarded these views, because they had been too indefinitely stated, but it certainly carried the negative too far, when it assumed that the causes of cataract are entirely unrecognized. It appeared to be determined that only one disease favored the development of cataract, namely—diabetes mellitus; in general we contented ourself in the majority of cases to look upon cataract as the expression of old age.

About eight years ago I treated a laborer, 50 years of age, suffering from a severe iritis, associated with considerable opacity of the aquous humor. The case recovered without any synechia and with complete subsidence of opacity. Five months later I again saw the patient presenting a cataract in the completely restored eye at the time of his discharge. The appearance of this form of vision disturbance, which I observed before and after a number of cases, which had been preceded either by an abscess of the cornea and simultaneous opacity of the aquous humor; an iritis, or an opacity of the vitrious humor, influenced me in bestowing the most careful attention to the pathognomy of cataract.

Should I express the result of my observations in a few words, I could state them in this hypothesis; that the formation of cataract is never a primary but always a secondary phenomenon. Its appearance is only made possible by certain inflammatory or atrophic changes in the sphere of the uveal tract. No processes of disease of the nervous opticus, or of the retina, unassociated with changes of the vitrious body, exert an influence upon the development of cataract. The old position, that rheumatism, athritis, syphilis, etc., lays the foundation to the development of cataract, finds accordingly its confirmation, but only in that sense, as these or other forms of disease give an impulse to pathological changes in one or the other part of the uveal tract. The key to the success or non success of many operations for cataract, I was only enabled to find, as time had taught me, to properly estimate these pathological disturbances and to seek in them the factors which as well introduce the formation of cataract, as they also determine, in coalition with technics, the result of the operative closing scene.

The most favorable forms of cataract are the so-called age cataract; their gradual development and the hardness of the lens, permit them to appear rather as the product of senile changes than as the result of pathological disturbances in the back-ground of the eye. Very often these forms are stationary, especially when they are associated with narrow, radiating lines of opacity. I can re-call cases, which nine and ten years ago, were standing upon the same degree of development upon which they are standing this day.

A developing cataract, with dense opacity of the lens, gains from that moment a softer consistence in its peripherical strata and advance unproportionally more rapid, if an acute appearing pernicious influence should have attacked any part of the globe of the eye, be it either in the form of a slight jar, or due to the influence of a transient opacity of the vitrious humor.

A formation of cataract which results after iritis or abscessus cornea under the influence of a pathological changed aquous humor presents a hard coherent but never a radiating conformation. It appears that this fact stands in connection to the observation, that a soft cataract exposed by division to the influence of the aqueous humor, progresses steadily in its absorption only so long as the iris remains free from inflammation. So soon as through the accompanying iritis an altered fluid of the eye is secreted, the absorption ceases, and the previously soft lens becomes dense.

Should a rapid formation of cataract takes place, after separation of the retina (by effusion or other causes,) then the consistence of the same is soft, probably because through displacement of the normal consistence of the vitrious humor a more abundant secretion of the watery constituents has been established. From that moment, however, in which a re-action on the part of the inflamed iris takes place, or that the vitrious humor becomes the seat of diseased transudations, the lens ceases to be soft, and attains a great density throughout all its parts. A cataracta accreta hardly ever occurs as a soft formation, not because this form is generally only observed in the advanced periods of life, but because its consistence is the product of the development of a diseased vitrious humor.

A child aged four months, that was born blind, through fœtal irido-choroiditis, the result of secondary syphilis, presented as a complication, a hard, yellowish appearing cataract. The same hard form I also observed in a child 17 months old. It is true that the forms of cataract occurring during the period of childhood are soft, nevertheless I have seen the yellow, waxy formation of cataract with an unusual tough nature in a boy eight years old, who, after previously performed iridectomy upon both eyes, was subjected to division of the lens. Notwithstanding the greatest precaution, a purulent choro-iritis became developed, which resisted all remedies, and finally ended in the complete destruction of the eyes.

Viewed from a purely technical stand-point, such a result is absolutely unaccountable, and it only then receives its proper explanation when we look upon the waxy cataract as the expression of a latent choro-iritis, which may assume the most dangerous dimensions upon any operative interference. All forms of cataract which can be traced back to a previous cyclitis or an acute developed glaucoma, are distinguished by their hard consistence. When in general the maxim is true that rapid formations of cataract favors a soft consistence, those resulting after cyclitis and glaucoma must be excluded. Upon several occasions I observed the formation of the hardest forms of cataract within a few weeks, without noticing radiating lines at any one time. Again a developing radiating cataract would receive a different conformation when an inter-current complication of acute glaucoma presented itself. When we take all these circumstances into consideration, we are involuntarily forced to the conclusion that the character of the developing cataract depends more or less upon the preceding disturbance of vision.

That diabetes mellitus pre-disposes to the formation of cataract is a long recognized fact. It appears in this form only as the prototype of a series of disturbances which are capable of being carried back to a great poverty of the blood as a general and original cause. Seldom only was a posterior polar opacity of the lens observed as the point of origin of a cataract formation, the rule being that only a trifling, or at least a very moderate nucleus is formed, the substance of the lens having undergone a half soft and half hard transformation, the color always being a shade into the bluish white. The concurrence of these phenomena is so constant that I have accustomed myself to see in them the expression of marasmus. The occurrence of this form I observed mostly attacking the middle-aged, after exhausting metrorrhagia, expectoration of blood, poor and insufficient food, as well also as after depressing mental influences; it appears that the loss of the nutritive blood-elements which the organism has sustained, leads to an absorption of the watery constituents, which not only exert an influence upon the formation but also upon the consistence of the lens.

Young women, who, during the period of lactation, are attacked by a transient transudation of the vitrious body, are especially exposed to posterior polar cataract. To the same processes also are exposed firemen, puddlers, and all such who in the nature of their occupation are exposed to the influences of an intense fire, which not only exerts a dazzling but also a congestive influence upon their eyes.

After slight contusions and concussions which the ball of the eye had received through the intervention of a foreign body, I could trace in ten or twelve cases the rapid formation of a posterior polar cataract, notwithstanding complete integrity of the capsule of the lens. It appears that by such traumatic occurrences the connective relations between the lens and the cup-like depression in the vitrious humor becomes somewhat loosened, and by the relative destruction of the nutrition of the lens, the character of the cataract is stipulated. A few months ago I saw a miner, who, while at work in the shaft, felt a dull pain, with sudden obscuration of vision, upon the thus far excellent left eye—phenomena which could only be attributed to effusions of blood into the vitrious body.

At the first examination, occurring three weeks subsequently, the seat of hemorrhage could no longer be established, but in connection with slight opacity of the vitrious humor there existed an opacity of the capsule involving two-thirds of the posterior wall of the lens. The trouble remained stationary; subsequently I adopted artificial ripening of the cataract; restoration of vision resulted in a few weeks after extraction.

There exists no doubt that the condition of the vitrious humor is of the greatest importance for the transparency of the posterior pole of the lens. All processes of atrophy occurring in the choroidea as they appear in the form of retinitis pigmentosa, selerotica-choroiditis posterior, choroiditis disseminata, necessarily also involve an obliteration of the nutritive network 'of the vitrious body. It therefore cannot be due to accident when the above mentioned diseased processes favor the development of cataracta polaris posterior. In eighty cases of retinitis pigmentosa I observed this complication twenty times upon both sides, and twice upon one side; furthermore, seventeen times with choroiditis disseminata and unusually frequent with sclerolico-choroiditis posterior. It is noticable that this form of cataract when associated with retenitis pigmentosa, remains in the majority of cases upon a very low stage of development, having during the whole period of my practice observed but one instance of developed radiating cataract occurring in the person of an aged female peasant. The diagnosis of co-existence of retinitis pigmentosa was regarded probable prior to the operation. The successful extraction of the cataract from both eyes gave to the patient an exceedingly imperfect, hardly mentionable power of vision, but to me the opportunity of an opthalmoscopic examination of the back part of the eye.

When contrary to our prognosis polar conformations of long duration, assume an unexpected rapid course, it is safe to assume with great certainty that inter-current opacity of the vitrious humor has become established. In a woman, near forty years old, I saw a cataracta polaris posterior complicated by retenitis pigmentosa, which for many years had remained stationary, rapidly increase double in circumference as opacity of the vitrious humor became developed without any traceable cause. The same condition I could establish twice with choroiditis disseminata. When we observe in connection with sclerotica choroiditis posterior, infrequently a stand-still, but generally a rapid development, it is probably to be attributed to the circumstance, that the vitrious humor is here more frequently exposed to opacity on account of the hidden anomalies in the choroid, and thus more readily exert an unfavorable influence upon the nutiition of the lens.

